# Stronger pack warnings predict quitting more than weaker ones: finding from the ITC Malaysia and Thailand surveys

**DOI:** 10.1186/1617-9625-11-20

**Published:** 2013-09-18

**Authors:** Ahmed I Fathelrahman, Lin Li, Ron Borland, Hua-Hie Yong, Maizurah Omar, Rahmat Awang, Buppha Sirirassamee, Geoffrey T Fong, David Hammond

**Affiliations:** 1 Clearing House for Tobacco Control, the National Poison Center, Universiti Sains Malaysia, Minden, Pulau Pinang 11800, Malaysia; 2 VicHealth Centre for Tobacco Control, Cancer Council Victoria, 1 Rathdowne Street, Carlton, Vic 3053, Australia; 3 Institute for Population and Social Research, Mahidol University, Salaya, Thailand; 4 Department of Psychology, University of Waterloo, 200 University Avenue West, Waterloo, ON, Canada; 5 Department of Health studies & Gerontology, University of Waterloo, 200 University Avenue West, Waterloo, ON N2L 3G1, Canada; 6 Department of Pharmacy Practice, College of Pharmacy, Qassim University, P.O. Box 6800, Buraidah 51452, Saudi Arabia

**Keywords:** Cigarette package warning labels, Health knowledge, Quitting behavior, Malaysia, Thailand

## Abstract

**Background:**

We examined the impact of cigarette pack warning labels on interest in quitting and subsequent quit attempts among adult smokers in Malaysia and Thailand.

**Methods:**

Two overlapping cohorts of adults who reported smoking factory- made cigarettes from Malaysia and Thailand were interviewed face-to-face (3189 were surveyed at baseline and 1781 re-contacted at Wave 2; 2361 current smokers were surveyed at Wave 2 and 1586 re-contacted at Wave 3). In Thailand at baseline, large text only warnings were assessed, while at Wave 2 new large graphic warnings were assessed. In Malaysia, during both waves small text only warnings were in effect. Reactions were used to predict interest in quitting, and to predict making quit attempts over the following inter-wave interval.

**Results:**

Multivariate predictors of “interest in quitting” were comparable across countries, but predictors of quit attempts varied. In both countries, cognitive reactions to warnings (adjusted ORs; 1.57 & 1.69 for Malaysia at wave 1 and wave 2 respectively and 1.29 & 1.19 for Thailand at wave 1 and wave 2 respectively), forgoing a cigarette (except Wave 2 in Malaysia) (adjusted ORs; 1.77 for Malaysia at wave 1 and 1.54 & 2.32 for Thailand at wave 1 and wave 2 respectively), and baseline knowledge (except wave 2 in both countries) (adjusted ORs; 1.71 & 1.51 for Malaysia and Thailand respectively) were positively associated with interest in quitting at that wave. In Thailand only, “cognitive reactions to warnings” (adjusted ORs; 1.12 & 1.23 at wave 1 and wave 2 respectively), “forgoing a cigarette” (adjusted OR = 1.55 at wave 2 only) and “an interest in quitting” (adjusted ORs; 1.61 & 2.85 at wave 1 and wave 2 respectively) were positively associated with quit attempts over the following inter-wave interval. Salience was negatively associated with subsequent quit attempts in both Malaysia and Thailand, but at Wave 2 only (adjusted ORs; 0.89 & 0.88 for Malaysia and Thailand respectively).

**Conclusion:**

Warnings appear to have common mechanisms for influencing quitting regardless of warning strength. The larger and more informative Thai warnings were associated with higher levels of reactions predictive of quitting and stronger associations with subsequent quitting, demonstrating their greater potency.

## Background

More than 80% of the world smokers live in low and middle income countries [1]. In some developed countries, tobacco use is declining as a result of decades of tobacco control efforts and educational activities [1], including through high taxes, anti-smoking advertisements, media campaigns, and warning labels on packs [2,3]. By contrast, progress is less overall in developing countries, with some showing increases in tobacco production and use [1,3]. Achieving more progress in tobacco control may require focusing on the strategies that are less expensive to adopt, as poorer counties, by definition have less resources to commit. The World Health Organization (WHO) Framework Convention on Tobacco Control (FCTC) obligates ratifying countries to implement broad comprehensive tobacco control policies including the placement of rotating health warnings on tobacco packaging covering at least 30% (but ideally at least 50%) of the package front and back [4]. Strong health warnings on cigarette packaging are the most cost-effective educational strategy [5], since the only cost is that of implementing the policy.

Pack warnings can be particularly effective as smokers are potentially exposed to the messages every time they want to buy or smoke a cigarette. Cigarette pack warning labels have been shown to increase smoking-related health knowledge [6,7], and health knowledge is positively associated with quit intentions [8].

There is now strong evidence that forgoing cigarettes (that is, choosing not to smoke a cigarette that one would normally have smoked) and reporting that health warnings trigger thoughts about the harms of smoking and/or quitting are both predictors of subsequent quitting, and that avoidance of the warnings is also associated with increased quitting, but mediated through the frequency of thoughts about the harms [9]. Perhaps counter intuitively, reported levels of avoidance of warnings and thoughts about the harms are positively associated. Furthermore these reactions that predict quitting are increased with larger and/or graphic warnings [10]. The warnings appear to have at least part of their effect by generating negative emotions such as fear and disgust, with these emotions being important in generating enough force to stimulate quit attempts [10,11]. Most of this evidence for positive effects of health warnings comes from developed, largely English speaking countries.

There are important gaps in knowledge of the predictive capacity of measures of health warning effects in developing countries. Yong et al. [12] have shown that the introduction of the new graphic warnings in Thailand were associated with increased levels of reporting noticing, and both cognitive and behavioral reactions to the warnings compared to the previous text-only warnings, the same kinds of reactions that have been prospectively associated with increased quitting activity in developed countries [9]. An earlier cross-sectional paper found that in Malaysia, smokers’ frequency of thoughts about the health risks of smoking and reporting forgoing cigarettes as a result of noticing warnings both significantly predicted an interest in quitting [13]. However, no prospective study has been conducted to explore if these variables prospectively predict actual quitting activity as they do in developed countries.

Malaysia and Thailand are neighboring countries and both have ratified the FCTC [3]. Thailand is predominantly populated by Buddhist Thais, while Malaysia has a majority of Muslim Malays and significant minorities of Chinese and Indians. Malaysia is relatively more economically advanced and urbanized compared to Thailand. Thailand’s tobacco control legislation is stronger and longer-standing, especially with regards to the cigarette package warning labels where at the time of the baseline survey they had large black on white text-only warnings covering 50% of the main sides of the pack and contained specific detail about diseases while Malaysia had a small, general, text-only warning on one side to the pack. However, Malaysia had conducted its first ever national mass media based anti-smoking campaign in 2004 before this study commenced, and Thailand only conducted its first comprehensive campaign in 2006, between the first two waves of the study, with efforts prior to that restricted to legislation and to local health education efforts.

In March 2005, just after the first wave of this study, Thailand introduced new graphic warning labels with six rotating messages, also covering 50% of both main faces of the pack. There was no change in Malaysian warning labels over the period of this study.

The objective of the present study was to evaluate and compare the impact of cigarette pack warning labels on both interest in quitting and quit attempts of adult smokers in Malaysia and Thailand. We conducted two replications in each country: In Thailand for interest in quitting, the first analyses were in relation to the text-only warnings, while the second was to the new graphic warnings. For quit attempts, one was of reactions to text-only warnings in Thailand predicting quit activity in a period during which similarly sized graphic warnings were introduced, and the second involved reactions to graphic warnings predicting in a period where graphic warnings were present during the entire period. The smaller Malaysian warnings were unchanged over the two replications, so predictors should be consistent.

## Methods

### Design

Predictive study using cross-sectional data to predict interest in quitting and longitudinal data to predict reported quit attempts over the following year or so. The study was designed to make comparisons between the two countries, focusing on the varying strengths of warnings within and between countries.

### Participants

Respondents from the International Tobacco Control Southeast Asia Survey (ITC-SEA survey) who reported smoking factory-made cigarettes (FMC) at the time of the survey, and for prediction of subsequent quit attempts, provided outcome data at the next wave. In Thailand, a total of 1370 FMC smokers were surveyed at wave 1 (early 2005) and 1018 of them were successfully followed up at wave 2 (2006) (retention rate = 74.3%); a total of 1018 current FMC smokers (not the same ‘1018’ as above; these 1018 current FMC smokers included 341 replenishments and 677 re-contacts) were surveyed at wave 2 and 803 of them were followed up at wave 3 (2008). In Malaysia, a total of 1819 FMC smokers were surveyed at wave 1 and 763 of them were successfully followed up at wave 2 (retention rate = 41.9%); a total of 1343 current FMC smokers (including 689 replenishments and 654 re-contacts) were surveyed at wave 2 and 783 of them were followed up at wave 3.

The ITC SEA Survey is of approximately 2000 respondents in each country each wave, but includes users of hand-rolled tobacco which either have no warnings, or different ones to those on factory made cigarettes, so these smokers were not included in the analyses. Respondents are recruited from adults who had smoked at least 100 cigarettes in their lifetime and smoked at least weekly at the time of recruitment. A detailed description of the sampling and study design of the ITC SEA survey has been reported elsewhere [14,15]. Briefly, the ITC SEA adult smoker survey employs a multistage clustering sampling procedure [14,15]. Smokers were mainly surveyed in face-to-face interviews using standardized questionnaire. The questionnaire was developed by an international team of experts on tobacco control, with different backgrounds [16]. The original questionnaire was prepared in English and translated and then back-translated, then checked by bilingual researchers. Survey interviews were conducted in English or Malay in Malaysia and in Thai in Thailand. From wave 2, the sample is replenished from the same sampling frame.

### Measures

The ITC SEA questionnaire covers a broad range of anti-tobacco health policies. Warning-related variables from the survey were smokers’ reports of frequency of: noticing the tobacco warning labels, reading or looking closely at them, thinking about health risk because of them (think-harm), thinking about quitting because of them (think-quit), stopping from having a cigarette when about to smoke one because of the warnings (forgo) (all on 4-point scales), and avoiding looking at the warnings (coded as a binary variable: Yes- no). For the purpose of the present analyses, the noticing and reading variables were combined into a single scale (salience), as were think-harm and think-quit (cognitive reactions), as was done by Borland et al. [9]. Seven knowledge questions:, whether smoking causes: stroke, impotence, lung cancer, lung decay, stained teeth, and premature ageing (all in smokers), and lung cancer in non-smokers, were summed, then divided into low “less than 5” and high “5-7” because the distribution was highly skewed. A range of demographic variables was also assessed: country, age, gender and rural/urban.

Outcome measures were: 1) “any interest in quitting” (plan to quit smoking sometime in the future versus no thoughts of quitting) in the same survey wave, and 2) reports of having made quit attempts over the following inter-wave interval.

### Statistical analyses

Multiple logistic regressions were used to test for possible predictive associations between warning-related variables and the two outcomes. All multivariate analyses were adjusted for demographic variables (age, gender and rural/urban). The analyses proceeded in two main steps, first each individual key predictor controlling for the sociodemographics, followed by a composite analysis in which all predictors were added simultaneously. The odds ratios with their 95% CI were computed for each predictor variable. A p value <0.05 was considered statistically significant. All analyses were conducted using Stata Version 10.1.

## Results

### Sample retention

There were some socio-demographic differences in retention rates for the longitudinal analyses. Followed-up respondents were more likely to be rural residents (p < 0.001, for both wave pairs in both countries) as were older respondents (p < 0.001, for both wave pairs in Thailand and the wave 1–2 participants in Malaysia). For wave 1–2 participants, in Malaysia, those retained were more likely to have low baseline knowledge of the harms of smoking (51.7% vs. 60.2% for drop-outs, p < 0.001); and in Thailand, those retained were more likely to “think about quitting because of the warning labels” at baseline (82.5% vs. 77.7% for drop-outs, p = 0.047). For wave 2–3 participants, in Malaysia, those retained were more likely to have high “cognitive reactions” at wave 2 survey (76.9% vs. 71.5%, p = 0.03); and in Thailand, those retained were more likely to report avoiding the warnings at wave 2 (53.4% vs. 45.1%, p = 0.03).

### Basic comparisons by country and wave

Table 1 portrays, by country, the baseline demographic characteristics as well as Wave 1 and 2 responses toward warning labels, quitting behaviors, and knowledge about smoking-related health risks among the two re-contact cohorts. Overall, Thai smokers were older than the Malaysian (p < 0.01). The proportions of the females and rural residents were greater among the Thais (p < 0.001). The characteristics of the two wave-based cohorts were similar, although the second was slightly older.

**Table 1:** Demographic characteristics, predictor variables and outcome variables among adult smokers in Malaysia and Thailand who were re-contacted at Waves 2 and 3

	**Malaysia**	**Thailand**	**P value for country comparison**
	**N = 763 for W1- W2 cohort^!;**	**N = 1018 for W1-W2 cohort;**	
	**N = 783 for W2- W3 cohort**	**N = 803 for W2-W3 cohort**	
**Demographics (baseline of the W1-W2 cohort)**			
Age [Mean age in years (SD)]	42.1 (14.1)	43.9 (12.5)	<0.01#
Gender [n (%)]			<0.001##
Male	742 (97.2)	952 (93.5)	
Female	21 (2.7)	66 (6.5)	
Residence [n (%)]			<0.001##
Rural	352 (46.1)	678 (66.6)	
Urban	411 (53.9)	340 (33.4)	
**Predictor variables**			
Warning labels salience [Mean (SD)] at W1*	3.1 (1.6)	3.5 (1.7)	<0.001#
Warning labels salience [Mean (SD)] at W2**	2.9 (1.7)	3.7 (1.6)	<0.001#
Cognitive responses toward warning label [Mean (SD)] at W1	2.5 (1.8)	3.6 (1.9)	<0.001#
Cognitive responses toward warning label [Mean (SD)] at W2	2.3 (1.7)	3.8 (2.0)	<0.001#
Stopping from having a cigarette because of warnings/forgoing [n (%)] at W1	326 (44.6)	476 (46.8)	NS##
Stopping from having a cigarette because of warnings/forgoing [n (%)] at W2	242 (32.1)	399 (50.0)	<0.001##
Avoid looking at warnings [n (%)] at W1	160 (22.1)	359 (35.3)	<0.001##
Avoid looking at warnings [n (%)] at W2	117 (15.6)	426 (53.4)	<0.001##
Knowledge scores [Mean (SD)] at W1	5.1 (1.9)	5.7 (1.4)	<0.001#
Knowledge scores [Mean (SD)] at W2	5.2 (1.8)	6.1 (1.4)	<0.001#
**Outcome variables**			
Any interest in quitting [n (%)] at W1	430 (57.3)	418 (41.1)	<0.001##
Any interest in quitting [n (%)] at W2	407 (52.2)	239 (29.8)	<0.001##
Quit attempts [n (%)] at W2	306 (40.1)	758 (74.5)	<0.001##
Quit attempts [n (%)] at W3	316 (40.4)	602 (74.9)	<0.001##

^For details of the samples, please see the methods section. !“W1” means “Wave 1” of the survey; this applies to the other waves.

#T-test results; SD, standard deviation; ##Chi-square test results.

*”Salience” is a combined variable (see the measures subsection); for Wave 1, the number of cases used to compute the salience variable (and the mean) is slightly smaller than the total N for W1-W2 cohort, due to “can’t say, unaware” or missing data. **Similarly, for Wave 2, the number of cases used to compute the variable is slightly smaller than the total N for W2-W3 cohort. This applies to other predictor and outcome variables in the Table.

Consistent with the size of the warnings, reactions to the pack warnings were stronger in Thailand than in Malaysia for all measures. Moreover, Thai smokers were significantly more knowledgeable about smoking-related health risks (p < 0.001) both at baseline and in the following year and a greater proportion (p < 0.001) reported quit attempts between survey waves even though less Thais reported an interest in quitting at each predictor wave (Table 1).

### Testing for by-country interactions

To explore the predictors of interest in quitting and prospectively of quit attempts, we tested separately the interaction between each predictor and “country” (Table 2). This revealed significant interactions in the prediction of “interest in quitting” (cognitive responses * country, both at waves 1 and 2). No significant by-country-interaction was found at wave 1 for predictors of “quit attempts”, however, most by-country-interactions for wave 2 were significant (Table 2). Accordingly, the data of the two countries was presented separately for all analyses (in Tables 3 and 4).

**Table 2:** Responses towards warning labels predicting interest in quitting in the same year, and follow up- year quit attempts among adult smokers from Malaysia and Thailand; results of the predictor variable by country-interactions

	**Any interest in quitting in same year**	**Quit attempts in follow up year**
	**By country**	**By country**
	**Adjusted OR ( *95% CI *)**^**a**^	**Adjusted OR ( *95% CI *)**^**a**^
Salience (scale) at w1* country	1.01 (0.89 – 1.13)^NS^	0.99 (0.87 – 1.12)^NS^
At w2	0.93 (0.82 – 1.06)^NS^	1.10 (0.96 – 1.26)^NS^
Cognitive responses at w1 (scale)* country	0.82 (0.72 – 0.93)**	1.02 (0.91 – 1.14)^NS^
At w2	0.80 (0.70 – 0.91)**	1.25 (1.11 – 1.40)***
Forgoing at w1* country	0.71 (0.47 – 1.07)^NS^	0.92 (0.60 – 1.40)^NS^
At w2	1.29 (0.82 – 2.05)^NS^	3.39 (2.11 – 5.43)***
Avoiding at w1* country	1.01 (0.64 – 1.58)^NS^	1.40 (0.87 – 2.26)^NS^
At w2	0.91 (0.55 – 1.52)^NS^	2.38 (1.39 – 4.09)**
Any interest in quitting * country	-	1.38 (0.89 – 2.12)^NS^
At w2	-	3.68 (2.16 – 6.29)***
Knowledge at baseline * country	1.06 (0.70 – 1.59)^NS^	1.02 (0.67 – 1.56)^NS^
At w2	1.15 (0.71 – 1.87)^NS^	1.17 (0.73 – 1.87)^NS^

^a^Logistic regression analysis for each interaction term “variable*country” was tested separately and adjusted for main effects. Reference category for country: Malaysia and for other variable, the lowest category; ^NS^Not significant; ** p < .01; *** p < .001.

**Table 3:** Responses toward warning labels predicting interest in quitting in the same year among adult smokers from Malaysia and Thailand

	**Any interest in quitting in same wave**			
	**Malaysia (N = 721 for W1; N = 770 for W2)**		**Thailand (N = 1018 for W1; N = 800 for W2)**	
	**Crude OR ( *95% CI *)**^**a**^	**Adjusted OR ( *95% CI *)**^**b**^	**Crude OR ( *95% CI *)**^**a**^	**Adjusted OR ( *95% CI *)**^**b**^
Salience (scale) w1	1.11 (1.01 – 1.22)**	0.95 (0.85 – 1.06)^NS^	1.11 (1.03 – 1.20)**	0.99 (0.92 – 1.09)^NS^
w 2	1.09 (1.01 – 1.19)*	0.92 (0.83 – 1.03)^NS^	1.04 (0.95 – 1.14)^NS^	0.89 (0.79 – 1.01)^NS^
Cognitive responses (scale) w 1	1.69 (1.52 – 1.87)***	1.57 (1.39 – 1.77)***	1.38 (1.28 – 1.48)***	1.29 (1.19 – 1.40)***
w 2	1.59 (1.45 – 1.76)***	1.69 (1.49 – 1.92)***	1.28 (1.17 – 1.39)***	1.19 (1.07 – 1.32)**
Forgoing w 1	3.24 (2.36 – 4.43)***	1.77 (1.20 – 2.61)**	2.22 (1.72 – 2.87)***	1.54 (1.16 – 2.05)**
w 2	2.30 (1.67 – 3.17)***	1.04 (0.67 – 1.61)^NS^	2.95 (2.14 – 4.07)***	2.32 (1.60 – 3.39)***
Avoiding w 1	1.19 (0.83 – 1.70)^NS^	0.85 (0.56 – 1.31)^NS^	1.21 (0.93 – 1.56)^NS^	0.89 (0.67 – 1.18)^NS^
w 2	1.29 (0.87 – 1.94)^NS^	0.89 (0.56 – 1.44)^NS^	1.23 (0.90 – 1.67)^NS^	0.86 (0.61 – 1.21)^NS^
Knowledge at baseline ^c^ w 1	1.73 (1.29 – 2.33)***	1.71 (1.19 – 2.44)**	1.98 (1.50 – 2.60)***	1.51 (1.13 – 2.02)**
w 2	1.58 (1.18 – 2.11)**	1.14 (0.81 – 1.61)^NS^	1.88 (1.27 – 2.77)**	1.34 (0.89 – 2.02)^NS^
Pseudo R2 Wave 1 predictors	0.173	0.075	0.021	0.043
Pseudo R2 Wave 2 predictors	0.128	0.065	0.016	0.098

^a^Simple logistic (bivariate analysis); ^b^Multiple logistic regression analysis adjusted for age, gender, and rural/urban; ^c^Reference category = “lower to average knowledge” (0–4 scores), ^NS^Not significant; * p < .05; ** p < .01; *** p < .001.

**Table 4:** Responses towards warning labels predicting follow up- year quit attempts among adult smokers from Malaysia and Thailand

	**Quit attempts in follow up wave**			
	**Malaysia (N = 750 at W2; N = 780 at W3)**		**Thailand (N = 1018 at W2; N = 803 at W3)**	
	**OR ( *95% CI *)**^**a**^	**Adjusted OR ( *95% CI *)**^**b**^	**OR ( *95% CI *)**^**a**^	**Adjusted OR ( *95% CI *)**^**b**^
Salience at w1 (scale)	1.04 (0.95 – 1.14)^NS^	1.01 (0.91 – 1.12)^NS^	1.04 (0.96 – 1.13)^NS^	0.99 (0.90 – 1.09)^NS^
At w2	0.93 (0.86 – 1.02)^NS^	0.89 (0.81 – 0.99)*	1.02 (0.92 – 1.12)^NS^	0.88 (0.78 – 0.98)*
Cognitive responses at w1 (scale)	1.19 (1.09 – 1.29)***	1.09 (0.98 – 1.23)^NS^	1.20 (1.12 – 1.29)***	1.12 (1.02 – 1.22)*
At w2	1.03 (0.95 – 1.11)^NS^	1.09 (0.97 – 1.22)^NS^	1.29 (1.18 – 1.39)***	1.23 (1.11 – 1.37)***
Forgoing at w1	1.81 (1.34 – 2.45)***	1.36 (0.96 – 1.96)^NS^	1.64 (1.23 – 2.19)***	1.22 (0.88 – 1.69)^NS^
At w2	0.78 (0.57 – 1.07)^NS^	0.69 (0.46 – 1.04)^NS^	2.59 (1.85 – 3.64)***	1.55 (1.01 – 2.32)*
Avoiding at w1	1.11 (0.77 – 1.58)^NS^	0.90 (0.61 – 1.32)^NS^	1.51 (1.11 – 2.06)**	1.37 (0.99 – 1.89)^NS^
At w2	0.67 (0.44 – 1.01)^NS^	0.71 (0.45 – 1.12)^NS^	1.41 (1.02 – 1.94)*	1.08 (0.74 – 1.57)^NS^
Any interest in quitting at w1	1.47 (1.09 – 1.99)*	1.15 (0.80 – 1.64)^NS^	1.94 (1.44 – 2.63)***	1.61 (1.17 – 2.23)**
At w2	1.02 (0.77 – 1.36)^NS^	1.02 (0.72 – 1.43)^NS^	3.68 (2.36 – 5.75)***	2.85 (1.79 – 4.53)***
Knowledge at baseline ^c^ w1	1.15 (0.85 – 1.54)^NS^	1.16 (0.84 – 1.61)^NS^	1.27 (0.95 –1.71)***	1.03 (0.73 – 1.41)^NS^
At w2	1.25 (0.94 – 1.68)^NS^	1.31 (0.95 – 1.81)^NS^	1.46 (1.02 – 2.09)*	1.05 (0.70 – 1.56)^NS^

^a^Simple logistic (bivariate analysis); ^b^Multiple logistic regression analysis adjusted for age, gender, and rural/urban; ^c^Reference category = “lower to average knowledge” (0–4 scores), ^NS^Not significant; * p < .05; ** p < .01; *** p < .001.

### Prediction of “interest in quitting” across the two countries

For “interest in quitting”, bivariate analyses indicated that for both countries, cognitive reactions (ORs; 1.69 & 1.59 for Malaysia at wave 1 and wave 2 respectively and 1.38 & 1.28 for Thailand at wave 1 and wave 2 respectively), “forgoing cigarettes” (ORs; 3.24 & 2.30 for Malaysia at wave 1 and wave 2 respectively and 2.22 & 2.95 for Thailand at wave 1 and wave 2 respectively) and “knowledge” (ORs; 1.73 & 1.58 for Malaysia at wave 1 and wave 2 respectively and 1.98 & 1.88 for Thailand at wave 1 and wave 2 respectively) were positively predictive of interest at both waves. Salience was also positively associated except for Thailand at wave 2 (ORs; 1.11 & 1.09 for Malaysia at wave 1 and wave 2 respectively and 1.11 for Thailand at wave 1), but avoidance was unrelated (Table 3). Multivariate analyses revealed similar effects in the two countries with “cognitive reactions” being positively predictive at both waves (adjusted ORs; 1.57 & 1.69 for Malaysia at wave 1 and wave 2 respectively and 1.29 & 1.19 for Thailand at wave 1 and wave 2 respectively). “Forgoing” was also positively predictive, except for Malaysia at wave 2 (adjusted ORs; 1.77 for Malaysia at wave 1 and 1.54 & 2.32 for Thailand at wave 1 and wave 2 respectively). Knowledge was predictive in both countries at wave 1 (adjusted ORs; 1.71 & 1.51 for Malaysia and Thailand respectively), but at wave 2 it had no multivariate effects (Table 3).

### Prediction of subsequent quit attempts across the two countries

The associations between predictor variables and subsequent quit attempts were more complicated (Table 4). In both waves in Thailand all predictors except salience were positively associated with subsequent quit attempts, but in the multivariate analyses, only “an interest in quitting” (adjusted ORs; 1.61 & 2.85 at wave 1 and wave 2 respectively) and “cognitive responses” (adjusted ORs; 1.12 & 1.23 at wave 1 and wave 2 respectively) were independent predictors in both cohorts, with “forgoing” being a significant predictor in the second cohort (adjusted OR = 1.55, 95% *CI*: 1.01-2.32) and “salience” a negative predictor in the same cohort (adjusted OR = 0.88, 95% *CI*: 0.78-0.98). By contrast in Malaysia, when treated independently only “cognitive reactions” (OR = 1.19, 95% *CI*: 1.09-1.29), “forgoing” (OR = 1.81, 95% *CI*: 1.34-2.45) and “an interest in quitting” (OR = 1.47, 95% *CI*: 1.09-1.99) were associated, but only in the first cohort. In the multivariate analyses for the first cohort however, there were no independent significant effects. In the second cohort in Malaysia nothing predicted making quit attempts bivariately, but in the multivariate analyses, salience was negatively associated with subsequent attempts (adjusted OR = 0.89, 95% *CI*: 0.81-0.99, p < 0.05).

## Discussion

This study found that the predictors of “an interest in quitting" were similar in the two countries across the two waves of prediction. However, there were marked differences in predictors of making quit attempts. In Thailand both “an interest in quitting” and several of the health warning reactions were significantly predictive of subsequent quit attempts in both waves, but for Malaysia the effects were weaker, and indeed for the Wave2-3 analyses none of the health warning variables had significant positive relationships with subsequent quitting and there was a marginal negative effect for warning salience. This contrasted with the Wave1-2 analyses where cognitive responses and forgoing contributed predictive variance (albeit not significant for either in the combined analyses). This difference in prediction occurred in a context where the warnings remained constant.

For Thailand, the predictors of interest in quitting were similar to those factors prospectively predicting quit attempts, and these are essentially the same as those found in the developed, largely English speaking countries [9] providing support for some universality in reactions to warning labels.

In interpreting the results it is important to keep in mind that the larger and more prominent Thai labels, both text-only and graphic, were significantly more salient than the Malaysian ones, and, of more importance, the cognitive and the behavioral responses toward them that predict subsequent quitting were also higher among Thai smokers [13]. Thus the stronger associations with outcomes in the Thai sample provide more evidence for the greater effectiveness of strong warnings.

In Malaysia, where the warnings remained weak throughout the study, the effects differed by wave, particularly for prediction of making quit attempts where a marginal effect on quitting between waves 1 and 2, unexpectedly disappeared in the second to third waves. The finding on interest in quitting where we replicated that aspect of our earlier work [12], were more similar, although at wave 2 we failed to find independent effects for forgoing and knowledge, suggesting a weaker overall relationship. It is possible that these differences in prediction are related to the levels of community activity around smoking either in the period before the predictors were measured or during the period when quit attempts were being assessed. In the months leading up to Wave 1 of the survey [17], Malaysia conducted its first ever comprehensive mass media campaign to discourage smoking and encourage quitting. This campaign, called Tak Nak (“Say No” to smoking) had high reach and stimulated a lot of quitting activity [18]. From around the time of Wave1 there was little community-based activity for the rest of the period covered by our surveys. It might be that for weak warnings, like those in Malaysia, there needs to be some other form of encouragement for quitting before the warnings act as effective triggers to make quit attempts. We know community campaigns can have a positive effect on quitting [19,20], so this is plausible, although we cannot rule out the possibility that at least some of the effects are a more direct effect of the community-based campaign on responses to the predictor measures, even though they refer specifically to the health warnings. Further, we have not found interactive effects by country in our previous studies in the developed countries, suggesting that the effects of the weak warnings in the US which are accompanied by strong public education, at least in some places, can still have an impact.

It is notable in this study, as in others, that in multivariate analyses, warning salience was either unassociated or negatively associated with quitting activity. We think this is because to be aware of the warnings, you need to be aware of cigarettes, and there appears to be a net effect of noticing anything to do with cigarettes triggering thoughts of smoking (the so-called ironic process) [21]. This means it is particularly important to have warnings that are strong enough to overcome this tendency by engaging countering thoughts about the undesirability of smoking. That is, mere presence of warnings is not enough, to the extent they are ignored (not avoided), they will have no effect.

The similar pattern of predictors of “quitting attempts” and “an interest in quitting” across the two pairs of waves in Thailand suggests that the graphic and text-only warnings operate in essentially the same way. The main difference is that the new warning labels tended to generate higher levels of these desirable reactions [12]. Once again it is the capacity of warnings to evoke action-oriented thoughts or behaviors that signals an increased subsequent likelihood of taking the more substantial act of attempting to quit.

In Thailand we found bivariate positive associations between reported avoiding the warnings and subsequent quit activity, but as in the developed countries [9], this disappeared in the multivariate analyses. These consistent findings suggest that avoidance is not a problem and that those who sometimes avoid the warnings, also tend to think about quitting more and thus are more likely to take quit-related actions over time. What better way to avoid thinking about the harms of smoking than to no-longer smoke and no-longer have to worry!

### Limitations

As a correlational study, we cannot rule out the possible causal role of some unmeasured third variable. The most plausible would be some other form of health education which may have stimulated the reactions either independent of or in interaction with the health warnings, something we have already postulated as possibly contributing to the different findings on quit attempts in the two waves in Malaysia. Such effects could also have contributed to the strengthening of reactions in Thailand in Wave 2, in particular, via the promotion of some of the harms of smoking in the mass media, an area where Thailand became more active after our first survey. We do not know to what extent this additional activity in Thailand would have affected our results. The finding of prediction from Wave 1 (before such activity in Thailand) to wave 2 outcomes (i.e. which could have been affected by intervening health education) makes it more likely that some of the effects are real and attributable to the warnings. It would be useful to attempt to more directly study the interactive effects of different sources of health information on both interest in quitting and quitting.

A further limitation of the present study was the high attrition rate among respondents in Malaysia. However, retention was good in Thailand, and there is nothing about the differences we found between the two countries that would suggest that the differential retention had any impact on the findings, but cannot absolutely rule it out. It should be stressed that loss to follow-up does not affect the relationships within individuals found here, only the likelihood of similar relationships being found among those not resurveyed, something we think unlikely given the consistency of the findings with other work.

## Conclusions

In conclusion, the stronger graphic Thai warnings were effective in promoting quit attempts among smokers. This is the first finding of prospective impact of health warnings on quitting outside developed countries, and largely replicates the effects found there. Our findings also suggest that, even the weaker Malaysian labels appear to have been capable of motivating some interest in quitting, but, of themselves, may not be potent enough to stimulate quit activity. The possibility that weak health warnings on packs may not be sufficient to translate increased interest in quitting into action requires replication as it was unexpected. Overall, the findings provide evidence for the universality of effects of health warnings. Warnings appear to work by evoking concern about the harms of smoking; just noticing them is not enough. Stronger warnings, by evoking more of these reactions, are thus more effective in encouraging smokers to try to quit.

## Ethics committee approval

The study was cleared by Institutional Review or Ethics Committees at University of Waterloo, Cancer Council Victoria, Universiti Sains Malaysia and Mahidol University, and Roswell Park Cancer Institute.

## Competing interests

The authors declare that they have no competing interests.

## Authors’ contributions

GF, DH and HY were involved in conceiving the study design and survey content. BS, MO and RA supervised data collection and quality control, and provided country-specific intelligence. AIF, LL, HY and RB all contributed to the analytical strategy. AIF, LL and RB contributed to drafting original manuscript. Most contributed to providing input and suggestions to drafts. All approved the manuscript for submission.
